# Dysglycemia and Cardiometabolic Risk: Pathophysiological Rationale and the Emerging Role of Nutraceuticals in Integrated Prevention

**DOI:** 10.3390/nu18050868

**Published:** 2026-03-08

**Authors:** Arrigo Francesco Giuseppe Cicero, Giovanni Scapagnini, Davide Grassi, Giuseppe Marazzi, Andrea Zanchè, Alessandro D. Genazzani, Roberta Scairati, Annamaria Colao

**Affiliations:** 1 Cardiovascular Medicine Unit, Heart, Chest and Vascular Department, IRCCS Azienda Ospedaliero-Universitaria di Bologna, 40138 Bologna, Italy; 2 Hypertension and Cardiovascular Risk Research Center, Medical and Surgical Sciences Department, Alma Mater Studiorum-University of Bologna, Sant’Orsola-Malpighi Hospital, 40138 Bologna, Italy; 3 Department of Medicine and Health Sciences, University of Molise, 86100 Campobasso, Italy; 4 Department MeSVA–UOC Internal Medicine-Center of Obesity, Nutrition and Cardio-Nephro-Metabolic Prevention, University of L’Aquila, 67100 L’Aquila, Italy; 5 IRCCS San Raffaele, 00163 Rome, Italy; marazzig@gmail.com; 6 Pescara Local Health Authority (ASL), 65100 Pescara, Italy; zancoandrea@hotmail.it; 7 Center for Endocrinological Gynecology, Department of Maternal, Infant and Adult Medical and Surgical Sciences, University of Modena and Reggio Emilia, 41124 Modena, Italy; algen@unimo.it; 8 Department of Clinical Medicine and Surgery, Section of Endocrinology, Diabetology, Andrology and Nutrition, University of Naples Federico II, 80131 Naples, Italy; roberta.scairati@unina.it (R.S.);

**Keywords:** dysglycemia, cardiometabolism, nutraceuticals, diabetes, diet, micronutrients, glucose, insulin, phytocomplexes

## Abstract

Dysglycemia represents an early and progressive stage of cardiometabolic disease, characterized by IR, metabolic inflammation, and increased cardiovascular risk. Its high prevalence and largely asymptomatic course often lead to diagnostic and therapeutic inertia, resulting in missed opportunities for early intervention. Recognizing dysglycemia as a disease continuum rather than a transitional condition supports the need for anticipatory and integrated preventive strategies. Within this framework, nutraceuticals are emerging as valuable supportive tools in the management of dysglycemia, particularly in individuals with increased metabolic risk who are not yet candidates for pharmacological therapy. Nutraceutical compounds can target key pathophysiological mechanisms underlying dysglycemia, including impaired insulin sensitivity, oxidative stress, chronic low-grade inflammation, and altered postprandial glucose metabolism. Clinical evidence supports the use of selected micronutrients, polyphenols, and standardized plant extracts in improving fasting and postprandial glycemic control. Phytocomplexes derived from plants such as *Mangifera indica*, *Momordica charantia*, and *Malus domestica* exert complementary and multitarget actions, including modulation of carbohydrate absorption, activation of AMPK-related pathways, enhancement of peripheral glucose uptake, stimulation of incretin secretion, and improvement of endothelial function. When integrated with lifestyle and dietary interventions, nutraceuticals may reduce glycemic variability, improve metabolic resilience, and delay progression toward type 2 diabetes. Overall, nutraceuticals represent a rational bridge between lifestyle measures and pharmacological treatment in the personalized management of dysglycemia.

## 1. Dysglycemia: Definition, Epidemiology, and Clinical Relevance

### 1.1. Dysglycemia as a Continuum of Cardiometabolic Risk

Dysglycemia represents a heterogeneous clinical condition located along a pathophysiological continuum between normoglycemia and type 2 diabetes mellitus (T2DM) and is associated with a progressive and measurable increase in cardiometabolic risk, already evident in the early stages. Evidence derived from cohort studies and meta-analyses indicates that so-called prediabetes is not a metabolically neutral condition but rather an early disease stage characterized by increased all-cause and cardiovascular mortality [1]. A meta-analysis that included over 10 million individuals demonstrated that subjects with fasting plasma glucose levels between 100 and 125 mg/dL have a significantly increased risk of all-cause and cardiovascular mortality compared with normoglycemic individuals. Notably, risk curves were substantially overlapping in subjects with fasting glucose > 100 mg/dL and >110 mg/dL, suggesting that even modest but persistent elevations in glycemia are clinically relevant [1].

Regarding microvascular complications, a meta-analysis conducted in prediabetic populations documented a prevalence of retinopathy of approximately 8%, a rate markedly higher than that observed in normoglycemic individuals and indicative of organ damage already present in the prediabetic phase [2,3].

Similarly, other microvascular and systemic complications have been linked to dysglycemia, including diabetic nephropathy [4], a major microvascular complication affecting approximately 30–50% of patients with T2D, and diabetic neuropathy, characterized by progressive nerve fiber loss and sensory dysfunction and linked to chronic hyperglycemia and microvascular injury [4]. Importantly, neural and vascular alterations may begin already in impaired glucose tolerance, suggesting that neuropathic damage can start during the dysglycemic phase.

A meta-analysis of cohort studies involving more than 90,000 individuals showed a 27% increased risk of peripheral arterial disease in subjects with dysglycemia compared with normoglycemic individuals [5,6]. The relationship between dysglycemia and heart failure has been further clarified by a meta-analysis including 15 cohort studies and over 9 million individuals, in which dysglycemia, even in the absence of overt diabetes, correlated with a 10–15% increased risk of heart failure [7]. Given the high prevalence of dysglycemia in the general population, this relative increase translates into a clinically relevant absolute impact. From a pathophysiological perspective, dysglycemia is early associated with systemic IR, compensatory hyperinsulinemia, and multisystem metabolic dysfunction. Chronic exposure to suboptimal glycemic levels promotes the accumulation of advanced glycation end products, increased oxidative stress, mitochondrial dysfunction, and activation of a chronic low-grade inflammatory state [8]. These mechanisms represent central drivers of early vascular damage and contribute to the increased cardiovascular risk observed already in the prediabetic phase. In this context, the traditional categorical distinction between normoglycemia, prediabetes, and diabetes progressively loses clinical relevance in favor of a quantitative and longitudinal assessment of metabolic risk. Dysglycemia should therefore be interpreted as a dynamic and often persistent condition that interacts with other cardiometabolic risk factors—such as atherogenic dyslipidemia, visceral obesity, metabolic dysfunction–associated fatty liver disease (MAFLD), and hypertension—thereby accelerating progression toward the cardio–reno–metabolic syndrome [9,10,11].

A further critical aspect is the frequently asymptomatic nature of dysglycemia, which contributes to substantial diagnostic and therapeutic inertia. Fasting glucose values between 100 and 110 mg/dL are often underestimated in clinical practice, despite evidence showing a significant increase in cardiovascular risk already at these levels. This underestimation leads to the loss of a crucial time window during which early interventions on lifestyle, diet, and nutraceutical supplementation may substantially modify the cardiometabolic risk trajectory [12,13,14].

Overall, these findings support the need to consider dysglycemia not as a simple transitional condition but as an early stage of cardiometabolic disease, already accompanied by subclinical organ damage and a quantifiable increase in adverse outcomes (Table 1).

### 1.2. Epidemiology of Dysglycemia

Dysglycemia represents a condition of very high prevalence in the general population and constitutes one of the main epidemiological drivers of global cardiometabolic risk. According to estimates based on the American Diabetes Association (ADA) criteria, the prevalence of impaired fasting glucose (IFG), impaired glucose tolerance (IGT), or both may affect up to 35–40% of the adult population, including undiagnosed individuals [18].

Epidemiological data indicate that a substantial proportion of individuals with dysglycemia remain undetected by healthcare systems due to the asymptomatic nature of the condition and the lack of systematic screening. Observational studies show that approximately 50% of individuals receive a diagnosis of T2DM when microvascular or macrovascular complications are already present, suggesting a prolonged period of silent exposure to an unfavorable metabolic environment [18,19]. A retrospective study that used data from the World Health Organization (WHO) reported that the healthspan–lifespan gap has widened worldwide in the past two decades [20], showing that, despite an increase in overall life expectancy, the burden of disease has increased. For example, the healthspan–lifespan gap in the U.S. grew from 10.9 years (2000) to 12.4 years, meaning people live more years with poor health. The same holds true in Italy, but with an even wider gap. Data from the Italian Institute of Statistics (ISTAT) on Equitable and Sustainable Well-Being (BES) indicate that, despite an average life expectancy of 83.4 years, healthy life expectancy is approximately 58 years, resulting in a gap exceeding 20 years (compared to 12.4 years for the US) [21]. This discrepancy reflects the high burden of non-communicable chronic diseases, largely attributable to metabolic disorders, including dysglycemia, obesity, and metabolic syndrome.

Analysis of behavioral determinants reveals a heterogeneous pattern. Over the past decade, the prevalence of sedentary behavior has shown a significant reduction, while obesity rates have remained substantially stable. In contrast, a marked deterioration in dietary quality has been observed: adequate consumption of fresh fruit has declined from 22% to 16% of the population, indicating a reduction in the intake of fiber, micronutrients, and bioactive compounds [21]. These data are consistent with evidence from the Global Burden of Disease (GBD) study, which identifies dietary factors as major determinants of type 2 diabetes risk. Most diet-attributable risk derives from nutritional deficiencies—such as fiber, fruit, vegetables, flavonoids, and micronutrients—rather than from caloric excess [12,13,14]. This aspect has relevant preventive implications, as it suggests that qualitative improvements in dietary patterns may significantly reduce the prevalence of dysglycemia.

From a demographic perspective, the progressive aging of the population further amplifies the epidemiological burden of dysglycemia. Population-based studies show that the prevalence of glycemic alterations increases exponentially after the age of 50, in parallel with increases in IR, visceral obesity, and MAFLD. However, more recent data indicate an increasing diffusion of dysglycemia also among younger age groups, in association with overweight, sedentary behavior, and dietary patterns characterized by a high consumption of ultra-processed foods [18].

Overall, these findings delineate dysglycemia as an epidemiologically pervasive condition. Its high prevalence, combined with the strong association with cardiovascular events and mortality, makes the development of primary and secondary prevention strategies a priority, aimed at early identification of individuals at risk and the implementation of effective lifestyle and nutritional interventions.

### 1.3. Dysglycemia, Insulin Resistance, and Reproductive Health

Dysglycemia and insulin resistance (IR) exert clinically relevant effects on reproductive and hormonal health, reflecting the close interaction between glucose metabolism, endocrine regulation, and vascular function. A sex-oriented approach to cardiometabolic prevention is increasingly recognized as necessary, since metabolic risk follows distinct biological and clinical trajectories in women and men, with specific windows of vulnerability across the life course. Within this framework, reproductive disorders may represent early clinical manifestations of systemic metabolic dysfunction, anticipating the development of overt cardio–reno–metabolic disease [22].

#### 1.3.1. Insulin Resistance as a Pathological Phenomenon in Women

Insulin resistance does not exclusively represent a pathological condition but may also occur as a physiological adaptation during specific phases of female life. Three critical phases can be identified: (i) puberty, during which a transient increase in IR supports somatic growth and sexual maturation [23]; (ii) pregnancy, characterized by physiological maternal and fetal IR, functional to fetal energy supply [24]; and (iii) menopause, during which the decline in estrogen levels is associated with increased IR, redistribution of adipose tissue toward the visceral compartment, and a worsening cardiometabolic profile [25]. These physiological adaptations may evolve into persistent IR in the presence of environmental, nutritional, and genetic–epigenetic predispositions. Among these, particular relevance is attributed to a family history of diabetes and to being born small for gestational age (SGA) or with intrauterine growth restriction (IUGR), conditions linked to intrinsic IR and increased risk of dysglycemia already at a young age [26].

#### 1.3.2. Insulin Resistance and Gynecological Disorders

In the gynecological setting, IR represents a common pathophysiological driver of several clinical conditions. Among these, polycystic ovary syndrome (PCOS) constitutes the most representative paradigm. Epidemiological studies indicate that 60–75% of overweight or obese women with PCOS and 18–30% of normal-weight women with PCOS exhibit IR, while up to 85% of patients present overweight- or obesity-associated compensatory hyperinsulinemia [27,28,29]. Hyperinsulinemia amplifies ovarian androgen production, alters granulosa cell function, and contributes to ovulatory dysfunction, anovulation, and infertility. Although IR is not included among the Rotterdam diagnostic criteria, it is currently recognized as one of the main pathogenetic mechanisms of PCOS and a key determinant of long-term cardiometabolic risk [30].

#### 1.3.3. Menopause and Cardiometabolic Risk

With the menopausal transition, the reduction in estrogen levels leads to an acceleration of metabolic alterations, with increased IR, redistribution of adipose tissue toward an “android” phenotype, worsening of glycemic and lipid parameters, and a significant increase in cardiovascular risk [31]. Women with a history of PCOS show significantly more pronounced glycemic and insulin responses during the oral glucose tolerance test (OGTT) in the postmenopausal period compared with the general population, thereby configuring a higher cardiometabolic risk profile [31].

#### 1.3.4. From Fertility to Cardiovascular Prevention

In clinical practice, women frequently access gynecological care for reproductive issues (e.g., menstrual irregularities, infertility, and ovulatory disorders), while underlying glycometabolic alterations often remain unrecognized. Early identification of dysglycemia and IR may improve ovulatory regularity and reproductive outcomes, reduce the risk of gestational diabetes and obstetric complications, prevent progression toward T2DM, MAFLD, and cardiovascular disease, and reduce the cardiometabolic burden in postmenopausal women, particularly when hormone replacement therapy is not feasible or not accepted. In this context, dysglycemia assumes the role of an early marker of systemic vulnerability, particularly relevant in the female population, in which cardiovascular risk has historically tended to be underestimated [22]. Management of IR and dysglycemia in women is primarily based on lifestyle interventions. However, the complexity of the underlying pathophysiology and the prolonged duration of risk exposure often require additional supportive strategies. Targeted nutritional and nutraceutical interventions may therefore represent a rational complement, particularly in women with PCOS, overweight, menopausal transition, or a history of gestational diabetes [31].

In addition to nutrient composition, emerging evidence suggests that the timing of food intake and circadian alignment may influence both metabolic and reproductive outcomes (Box 1).

Box 1 **Chrononutrition and Dysglycemia: Aligning Meal Timing with Metabolic Rhythms.**

Chrononutrition refers to the alignment of food intake with endogenous circadian rhythms, which regulate glucose metabolism, insulin sensitivity, lipid metabolism, and energy expenditure. Growing evidence indicates that not only what is eaten, but also when food is consumed, represents a relevant determinant of metabolic homeostasis and cardiometabolic risk. Glucose tolerance, insulin sensitivity, and pancreatic β-cell function follow a circadian pattern, being more efficient during the early hours of the day and progressively reduced during the evening and night. Disruption of this temporal architecture—through late meals, irregular eating patterns, or concentration of caloric intake in the evening—has been associated with increased IR, impaired postprandial glycemic control, and greater glycemic variability, even in the absence of overt diabetes [32,33].

Observational and interventional studies indicate that concentrating carbohydrate intake in the earlier part of the day improves postprandial glycemic response, reduces insulin excursions, and promotes synchronization of peripheral metabolic clocks. Conversely, skipping breakfast or shifting caloric intake toward the evening plays a significant role in worsening glycometabolic profiles and increasing the risk of progression from dysglycemia to T2DM [32]. Evidence-based practical recommendations include avoiding breakfast skipping, limiting late evening meals, distributing carbohydrate intake predominantly during daytime hours, and integrating dietary patterns rich in bioactive compounds that support circadian metabolic regulation [33]. In individuals with dysglycemia, insulin resistance, or increased cardiometabolic risk—particularly in women undergoing the menopausal transition—chrononutrition may represent a tool for personalized prevention, acting synergistically with dietary quality, physical activity, and nutraceutical support.

#### 1.3.5. Dysglycemia and Male Sexual Health

The reproductive consequences of dysglycemia are not limited to women. In men, dysglycemia has been linked to sexual dysfunction, particularly erectile dysfunction (ED), which is increasingly recognized as an early marker of endothelial impairment and cardiometabolic vulnerability. A meta-analysis including nine observational studies (n = 10,980 men) showed that, compared with normoglycemic subjects, men with prediabetes had a significantly higher prevalence of ED (OR 1.62; 95% CI 1.28–2.07), with a stronger association reported in studies including younger men (mean age < 50 years) [34]. These findings support the interpretation of ED as a clinical manifestation of early dysglycemia-related vascular dysfunction, driven by oxidative stress, inflammation, and impaired endothelial nitric oxide signaling, which are also central mechanisms in the development of atherosclerosis and cardiovascular disease.

## 2. Nutraceuticals and Dysglycemia: Biological Rationale and Clinical Evidence

Nutraceuticals represent a class of bioactive compounds and standardized phytocomplexes with documented biological activity on metabolic and vascular pathways relevant to cardiometabolic disease. In the context of dysglycemia, their interest lies in the ability to modulate core pathophysiological mechanisms—including IR, oxidative stress, chronic low-grade inflammation, mitochondrial dysfunction, endothelial impairment, and postprandial glucose variability—already active in the early phases of the metabolic continuum [35,36,37].

Unlike single-target pharmacological agents developed for established diseases, nutraceuticals are typically characterized by multitarget and pleiotropic actions, making them conceptually suitable for early intervention and metabolic risk modulation in subjects with subclinical or borderline alterations. A wide range of bioactive compounds have been investigated for the management of prediabetes and dysglycemia, including plant extracts, micronutrients, marine-derived compounds, and phytochemical complexes (see comprehensive overview in [38]).

However, not all investigated compounds demonstrate the same level of clinical robustness. As summarized in Table 2, several agents show substantial heterogeneity in dosing regimens and extract standardization, frequent reliance on multi-ingredient formulations that limit mechanistic attribution, short study duration, and, in some cases, tolerability or safety concerns. For these reasons, we selected nutraceuticals supported by comparatively stronger mechanistic rationale and consistent clinical evidence. The nutraceutical compounds discussed in the following sections illustrate a mechanistically integrated approach to dysglycemia management, as they target complementary and potentially synergistic pathways—such as insulin secretion and sensitivity, AMPK activation, modulation of postprandial carbohydrate absorption, incretin stimulation, oxidative stress reduction, and endothelial function improvement. These mechanisms collectively address the multidimensional nature of dysglycemia along the cardio–reno–metabolic continuum.

### 2.1. Micronutrients and Bioactive Compounds with Evidence on Glucose Metabolism

#### 2.1.1. Vitamin D

Vitamin D deficiency is associated with increased systemic inflammation and greater IR, supporting its potential relevance in subjects with dysglycemia and increased cardiometabolic risk [59], playing a direct role in the regulation of glucose metabolism through multiple biological mechanisms (Table 3). Vitamin D receptors (VDRs) are expressed in pancreatic β-cells, which also possess the enzymes required for its hydroxylation [60] and whose dysfunction is associated with worsening of glycemic control [61]. Moreover, the promoter region of the insulin gene contains a vitamin D–responsive element (VDRE), supporting an involvement of vitamin D in the regulation of insulin secretion [59,62].

Randomized controlled trials (RCTs) suggest a modest benefit of vitamin D supplementation in reducing progression from prediabetes to type 2 diabetes mellitus (T2DM). The Tromsø (n = 511) [63], D2d (n = 2423) [73], and DPVD (n = 1256) [64] trials reported hazard ratios (HRs) for incident T2DM of 0.90 (95% CI 0.69–1.18), 0.88 (95% CI 0.75–1.04), and 0.87 (95% CI 0.67–1.17), respectively. Although individually non-significant and smaller than the anticipated 25–30% risk reduction, effects were directionally consistent.

An individual participant data meta-analysis (n ≈ 4200) showed a significant 15% reduction in diabetes risk (HR 0.85; 95% CI 0.75–0.96) and a 30% increase in regression to normoglycemia (HR 1.30; 95% CI 1.16–1.46) [65]. Additional meta-analyses confirmed a ~10% reduction in progression and a 24% increase in reversion to euglycemia among individuals with prediabetes [66,74,75]. Higher achieved intratrial 25(OH)D levels were associated with lower diabetes risk [76], and recent data confirm increased regression to normoglycemia [77]. Importantly, the benefit appears to depend on baseline vitamin D status. No clear effect modification was observed at baseline levels < 20 ng/mL in one study [63], whereas post hoc analyses showed significant risk reduction only in participants with marked deficiency (<12 ng/mL) [65]. This supports the concept that correction of deficiency, rather than supplementation per se, drives metabolic benefit [78,79,80,81].

In summary, vitamin D supplementation in individuals with prediabetes is associated with modest reductions in progression to T2DM and increased regression to normoglycemia, with the strongest evidence observed in those with baseline vitamin D deficiency.

#### 2.1.2. Chromium

Chromium is an essential trace element involved in the maintenance of normal glucose homeostasis. A systematic review and meta-analysis of RCTs assessed the efficacy and safety of chromium supplementation in diabetes [68]. The meta-analysis included 25 RCTs, with a total sample size of approximately 1600 participants. Overall, chromium supplementation correlated with a significant reduction in fasting plasma glucose and a modest improvement in glycemic control, whereas effects on HbA1c were less consistent across studies (Table 3). Subgroup analyses suggested that benefits might be more evident in subjects with poorer baseline glycemic control, while data were insufficient to draw firm conclusions regarding differences by sex, ethnicity, or age, as these variables were inconsistently reported. Based on this evidence, the European Food Safety Authority (EFSA) has approved a health claim for chromium related to the maintenance of normal blood glucose levels, making it one of the few micronutrients with a formal indication in this field [82].

#### 2.1.3. Flavonoids and Antioxidant-Rich Foods

Across prospective and observational evidence, higher dietary intake of antioxidant-rich foods has been consistently correlated with lower cardiometabolic risk and mortality, although causality cannot be established. In the dose–response meta-analysis on dietary total antioxidant capacity (TAC) and dysglycemia, 10 observational studies (n = 170,919) reported that higher TAC correlated with reduced risk of prediabetes (RR 0.58; 95% CI 0.34–0.97) and diabetes (RR 0.71; 95% CI 0.58–0.87) [69]. Complementing these findings, a meta-analysis of 15 prospective cohort studies showed that higher flavonoid intake correlated with lower cardiovascular disease mortality (RR 0.86; 95% CI 0.75–0.98) and borderline lower all-cause mortality (RR 0.86; 95% CI 0.73–1.00), with nonlinear dose–response patterns [70]. A larger pooled analysis of 16 cohorts (n = 462,194; 23,473 deaths; follow-up 4.8–28 years) confirmed inverse associations with all-cause mortality (RR 0.87; 95% CI 0.77–0.99) and cardiovascular disease mortality (RR 0.85; 95% CI 0.75–0.97) [71].

With specific regard to incident type 2 diabetes, a meta-analysis of 8 prospective studies (10 cohorts; n = 312,015; 19,953 cases; follow-up 4–28 years) found that higher flavonoid intake correlated with a modest reduction in T2DM risk (RR 0.89; 95% CI 0.82–0.96), with evidence of a curvilinear dose–response relationship and consistent findings across most flavonoid subclasses [72]. Overall, these data suggest a coherent association between flavonoid-rich dietary patterns and reduced cardiometabolic risk. However, all analyses are based on observational designs, rely on self-reported dietary intake, and are subject to residual confounding and heterogeneity in flavonoid databases and exposure assessment.

### 2.2. Phytocomplexes in the Management of Dysglycemia

In recent years, a growing body of experimental and clinical evidence has documented the role of specific plant extracts in the modulation of glucose metabolism and cardiometabolic risk. Unlike single micronutrients, plant-derived phytocomplexes exert multitarget actions, simultaneously influencing intestinal carbohydrate absorption, insulin sensitivity, mitochondrial function, chronic low-grade inflammation, and postprandial glycemic response (Table 4) [83]. This approach is particularly relevant in the dysglycemic phase, characterized by early but already structured metabolic alterations, in which integrated modulation of pathophysiological pathways may yield clinically meaningful benefits.

#### 2.2.1. *Mangifera indica* (Mango)

*Mangifera indica* is a polyphenol-rich fruit providing xanthones (particularly mangiferin), flavonoids, carotenoids, and dietary fiber, compounds with documented antioxidant and metabolic activity [84,85]. Preclinical evidence from high-fat diet murine models demonstrated that dietary supplementation with freeze-dried mango (1–10%) for 8–10 weeks improved fasting glucose, reduced adiposity, and enhanced insulin sensitivity, partly through activation of AMPKα and SIRT1 pathways involved in mitochondrial bioenergetics and oxidative stress modulation, suggesting mechanistic overlap with “metformin-like” signaling [84,86].

Translational evidence in humans is increasingly available. A double-blind randomized placebo-controlled trial in overweight patients with hyperlipidemia (n = 97 completers) showed that supplementation with 150 mg/day of purified mangiferin for 12 weeks significantly improved triglycerides, free fatty acids, HDL cholesterol, and Homeostatic Model Assessment for Insulin Resistance (HOMA-IR), although no significant changes were observed in LDL cholesterol or fasting glucose [87]. A small uncontrolled pilot study in obese adults (n = 20) reported reductions in fasting glucose after 12 weeks of freeze-dried mango pulp, though the absence of a control group limits interpretability [88]. More robust evidence derives from randomized controlled trials in metabolically at-risk populations: in adults with prediabetes (n = 23 completers), daily consumption of fresh mango (~300 g/day) for 24 weeks significantly reduced fasting glucose, improved insulin sensitivity, stabilized HbA1c compared with controls, and showed favorable trends in body composition, although the study was limited by small sample size and baseline group imbalances [89]. Similarly, in individuals with overweight/obesity and chronic low-grade inflammation, mango consumption correlated with improved insulin sensitivity, supporting a potential role in early metabolic dysfunction [90].

Overall, current evidence suggests that mango-derived phytocomplexes may exert insulin-sensitizing and metabolic benefits, particularly in subjects with dysglycemia or increased cardiometabolic risk. However, available trials remain relatively small and heterogeneous in design, dosage, and duration, warranting confirmation in larger, long-term randomized studies.

#### 2.2.2. *Momordica charantia* (Bitter Melon)

*Momordica charantia*, traditionally consumed in Southeast Asian dietary patterns, contains a range of bioactive compounds—including saponins, triterpenoids, and momordicin—that contribute to its documented hypoglycemic properties. Preclinical and mechanistic studies indicate that its metabolic effects are mediated through multiple complementary pathways, including inhibition of intestinal α-glucosidase and α-amylase activity with consequent reduction in carbohydrate absorption, upregulation of GLUT4 expression via AMPK pathway activation, and stimulation of bitter taste receptors (TAS2Rs) on enteroendocrine cells, promoting the secretion of incretins such as GLP-1, peptide YY, and cholecystokinin [91]. These mechanisms collectively support improvements in postprandial glycemic control, peripheral glucose uptake, and insulin sensitivity.

Clinical evidence, although heterogeneous in formulation and dosage, suggests potential metabolic benefits. Controlled intervention studies in individuals with prediabetes have reported that supplementation with freeze-dried *Momordica charantia* fruit for 8–12 weeks results in significant reductions in fasting plasma glucose and insulin levels, together with improvements in HOMA-IR [92]. Furthermore, a recent meta-analysis including 25 randomized controlled trials conducted in subjects with prediabetes or T2DM demonstrated a significant reduction in IR and overall improvement in glycemic control compared with placebo or standard care [93]. Nevertheless, variability in extract standardization, sample size, treatment duration, and study quality across trials limits the strength of definitive conclusions.

Overall, current evidence supports a potential role for *Momordica charantia* as an adjunctive nutraceutical strategy in early dysglycemia and IR, pending confirmation from larger, well-standardized randomized trials.

#### 2.2.3. *Malus domestica* (Apple)

Apples are a relevant source of polyphenols, including flavan-3-ols, procyanidins, flavonoids, and dihydrochalcones, such as phloridzin, a molecule historically associated with the discovery of sodium–glucose transporters (SGLTs). Preclinical studies have demonstrated that apple procyanidins improve IR in ob/ob murine models by suppressing hepatic expression of pro-inflammatory cytokines, suggesting a direct role in the modulation of metabolic inflammation [94].

Human evidence is also available. In a randomized placebo-controlled intervention study, supplementation with apple polyphenols for 8–12 weeks reduced post-load glycemic response in individuals with high-normal or borderline fasting glucose levels, suggesting a potential benefit in early dysglycemia [95]. Additional randomized, double-blind, placebo-controlled trials reported significant reductions in fasting plasma glucose, serum uric acid, and biomarkers of vascular oxidative stress, accompanied by improvements in endothelial function, supporting a broader cardiometabolic effect beyond glycemic regulation [96]. Long-term observational evidence from a prospective meta-analysis including more than 228,000 participants showed that regular consumption of apples and pears correlates with a significantly reduced risk of incident T2DM, with evidence of a dose–response relationship [97].

Overall, current evidence suggests that apple-derived polyphenols may contribute to improved glucose handling and vascular function, although heterogeneity in formulations, doses, and study populations warrants further confirmation in larger and longer randomized trials.

#### 2.2.4. Biological Rationale for the Combination of Phytocomplexes

**Figure 1:**
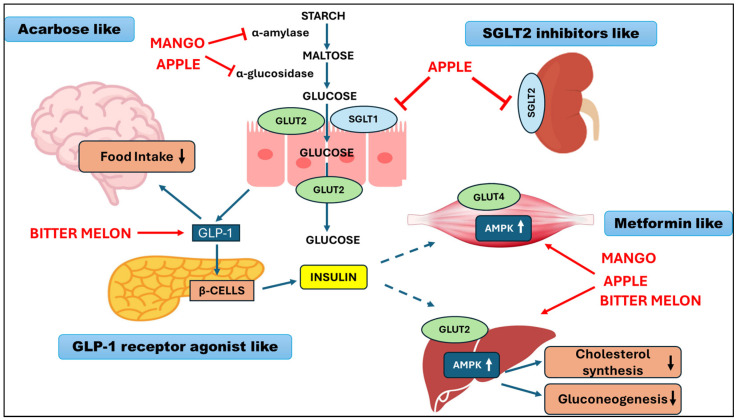
Schematic representation of the complementary mechanisms of action of *Mangifera indica* (mango), *Malus domestica* (apple), and *Momordica charantia* (bitter melon).

The combination of different standardized plant extracts represents a rational approach in the management of dysglycemia, as it allows coordinated intervention on multiple pathophysiological mechanisms involved in IR and early metabolic dysfunction (Figure 1). In this context, the combination of extracts derived from *Mangifera indica*, *Malus domestica*, and *Momordica charantia* appears particularly relevant due to the complementarity of their mechanisms of action. Extracts of *Mangifera indica*, rich in mangiferin, exert insulin-sensitizing effects through activation of AMPK and SIRT1 pathways, with subsequent improvement in mitochondrial function and cellular bioenergetics. These mechanisms are central to the regulation of glucose and lipid metabolism, particularly in the early stages of the dysglycemic continuum [85,87]. Apple polyphenols, including flavonoids, chlorogenic acid, and dihydrochalcones such as phloridzin, primarily act at the intestinal and vascular levels. These compounds modulate carbohydrate absorption through interaction with glucose transporters (particularly SGLTs), contribute to the reduction in postprandial glycemic response, and improve endothelial function, with potential benefits on cardiovascular risk [94]. *Momordica charantia* completes the profile of the combination through distinct but complementary mechanisms, including inhibition of the digestive enzyme α-glucosidase, increased GLUT4 expression in peripheral tissues, and stimulation of GLP-1 secretion mediated by activation of TAS2Rs at the enteroendocrine level. These effects contribute to improved insulin sensitivity, postprandial glycemic control, and satiety [91]. Experimental and clinical evidence suggests that the combination of these phytocomplexes may result in synergistic effects compared with individual extracts, including (i) greater inhibition of enzymes involved in starch digestion [88,90]; (ii) improved hepatic glucose uptake [15,87]; (iii) coordinated activation of AMPK–SIRT1–PGC-1α pathways, central to mitochondrial metabolism [59,68]; (iv) potential reduction in the overall glycemic index of meals [89,90]; and (v) integrated effects on insulin sensitivity, GLP-1 secretion, and appetite regulation [15,89].

These effects are of particular interest in the female population, in which IR represents a common pathophysiological node underlying several gynecological conditions. Modulation of IR may positively influence ovulatory function, hormonal profile, and cardiometabolic risk in women with PCOS, as well as in women who are overweight or undergoing the menopausal transition [28,29,30,31]. However, the combination of these bioactive compounds could be relevant to the male population too, where ED has been recognized as a clinical manifestation of early dysglycemia-related vascular dysfunction, driven by oxidative stress, inflammation, and impaired endothelial nitric oxide signaling [34].

## 3. Early Diagnosis and Intervention Strategies: The Role of the General Practitioner and the Patient

**Figure 2:**
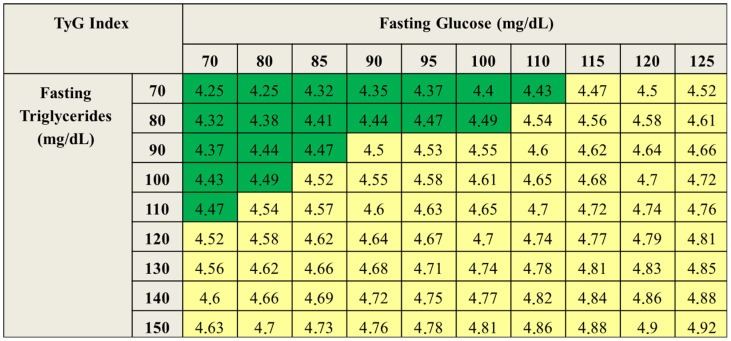
The triglyceride–glucose index (TyG index) is a parameter used to assess IR and cardiovascular risk. It is calculated as the logarithm of the multiplication of fasting triglyceride levels by fasting plasma glucose and dividing the result by two. A value below 4.5 is considered normal, whereas higher values indicate increased risk. Green colour includes values classified as normal, yellow colour the subobtimal ones.

Dysglycemia should be considered an early stage of the cardio–reno–metabolic continuum rather than a transitional condition between normoglycemia and T2DM. Even mild but persistent glycemic alterations are associated with increased cardiovascular and metabolic risk [2,7,9,10,11,12]. Early identification is therefore essential to prevent progression and organ damage. The general practitioner (GP) plays a central role in this process. As the first point of contact with the healthcare system, the GP is well positioned to detect subtle but persistent abnormalities—such as fasting plasma glucose values between 100 and 110 mg/dL, rising triglycerides, or unfavorable changes in body composition—which, although modest individually, define an increased cardiometabolic risk profile when considered collectively [7,8]. Reliance solely on diagnostic thresholds may delay intervention and contribute to clinical inertia [11,13]. In this scenario, the triglyceride–glucose index (TyG index; Figure 2) emerges as a simple, reproducible, and validated tool—also in the Italian population—capable of early identification of a condition of “persistent dysglycemia” and effective cardiovascular risk stratification [19]. The URRAH study, conducted in more than 16,000 individuals followed for 144 months, demonstrated that the TyG index shows an almost exponential relationship with all-cause and cardiovascular mortality, with threshold values around 4.5–4.6 capable of discriminating subjects at significantly increased risk [10].

Management in this phase should focus on risk modulation rather than categorical diagnosis. Lifestyle intervention remains the cornerstone of care. In selected individuals with increased cardiometabolic risk who are not candidates for pharmacological therapy, targeted nutritional approaches and evidence-based nutraceuticals may provide additional support by improving insulin sensitivity and glycemic control.

Patient involvement is equally important. Because dysglycemia is frequently asymptomatic, risk perception and adherence to preventive measures are often limited. Structured education is therefore necessary to translate laboratory findings into sustained behavioral change. Interventions recommended for cardiovascular prevention—high-quality diet, regular physical activity, weight control, and reduction in excess caloric and sodium intake—also reduce the risk of progression to T2DM. Attention should be given to dietary quality, meal timing and chrononutrition [32], and the adoption of Mediterranean or plant-based dietary patterns rich in bioactive compounds.

An integrated and longitudinal approach, based on early detection, lifestyle optimization, and, when appropriate, nutraceutical support, can prevent progression to T2DM.

### Nutraceuticals as a Bridge Between Prevention and Therapy

**Figure 3:**
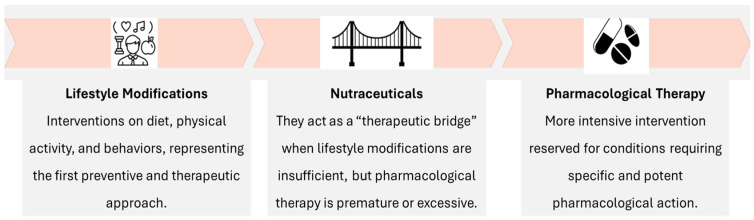
Nutraceuticals as a bridge between prevention and therapy.

Along the dysglycemia continuum, nutraceuticals are positioned as a therapeutic bridge between primary prevention and pharmacological treatment (Figure 3). Their rationale does not lie solely in glycemic control but rather in the ability to modulate key pathophysiological processes, including chronic low-grade inflammation, oxidative stress, endothelial dysfunction, IR, and mitochondrial dysfunction [83,98].

Clinical evidence indicates that several nutraceutical compounds can improve surrogate metabolic outcomes, such as fasting plasma glucose, insulin sensitivity indices, postprandial glycemic response, lipid profile, inflammatory markers, and endothelial function. However, the magnitude and consistency of these effects vary depending on the specific compound, formulation, baseline metabolic status, and study duration. In addition, many trials remain relatively short-term and rely mainly on intermediate endpoints rather than long-term clinical outcomes. Therefore, nutraceuticals should not be considered substitutes for pharmacological therapy but rather supportive interventions that may complement lifestyle measures [99]. Within this context, it is important to distinguish between micronutrients supported by authorized EFSA health claims—such as chromium, approved for the maintenance of normal blood glucose levels—and other bioactive compounds for which current evidence suggests potential preventive or metabolic-modulating effects, but without specific EFSA-authorized claims related to glycemic treatment.

Considering the available evidence, dysglycemia should be recognized as an early stage of cardiometabolic disease already associated with subclinical organ damage and a quantifiable increase in adverse events. The clinical goal is no longer to wait for the diagnostic threshold of diabetes to be reached but to anticipate intervention, shifting patients from the “grey zone” of latent risk toward proactive, personalized, and evidence-based management. An effective strategy should include (i) early identification through simple and reproducible markers (e.g., TyG index); (ii) structured lifestyle interventions; (iii) correction of nutritional deficiencies; (iv) targeted use of nutraceuticals; and (v) longitudinal monitoring of cardiometabolic risk. Only an anticipatory and integrated approach can truly prevent progression toward overt diabetes and its cardiovascular complications, helping to reduce the gap between life expectancy and healthy life expectancy.

## Figures and Tables

**Table 1:** Clinical outcome of dysglycemia, hyperglycemia, prediabetes and T2D.

Clinical Outcome	Population/Source	Risk Increase in Subjects with Dysglycemia
All-cause mortality	Meta-analysis of cohort studies (BMJ)	Prediabetes: ↑ significant risk already with fasting glucose >100 mg/dL; overlapping risk curves for >100 vs. >110 mg/dL [1]
Cardiovascular mortality	Meta-analysis of cohort studies	Prediabetes: ↑ significant risk compared with normoglycemic subjects [1]
Heart failure	Meta-analysis (15 studies, >9 million individuals)	Prediabetes: +10–15% risk [7,15]
Subclinical vascular damage	Observational and imaging studies	Hyperglycemia and T2D: increased silent atherosclerosis and endothelial dysfunction [9,10,11]
Peripheral arterial disease	Cohort studies (>90,000 individuals)	Prediabetes and T2D: ↑ risk compared with normoglycemic subjects [5,6,16]
Metabolism-related retinopathy	Meta-analysis in prediabetic populations	Prediabetes: ↑ prevalence in subjects with prediabetes [2,3]
Diabetic nephropathy/Diabetic kidney disease (DKD)	Comprehensive review	T2D: Affects ~30–50% of patients with T2D; leading cause of chronic kidney disease [4]
Diabetic neuropathy	Comprehensive review	T2D: Progressive loss of nerve fibers causing symptoms such as numbness/tingling/burning pain; vascular/nerve fiber changes can begin in IGT [4]
Cancer (overall and selected sites)	Meta-analysis of 16 prospective cohorts	Prediabetes: ↑ overall cancer risk (RR 1.15; 95% CI 1.06–1.23). Increased risks reported for stomach/colorectum, liver, pancreas, breast, and endometrium [17]

**Table 2:** Active compounds used for the management of prediabetes for which some issues related to dosage, tolerability or formulation have emerged.

Active Compound	Dosage Issues	Tolerability/Safety Concerns	Formulation Issues	References
Cinnamomum (cassia/verum/aromaticum)	Very wide dose range (120 mg–12 g/day); heterogeneous preparations (powder, aqueous extract, tea); lack of standardization.	Cassia contains high coumarin (0.8–10.63%) with potential hepatotoxicity at high doses.	Botanical variability (cassia vs. Ceylon); extract-dependent effects.	[39,40,41,42,43]
Berberine (berberine HCl)	Typical dose is 1–1.5 g/day; used alone or as an add-on; bioavailability-dependent formulations.	GI adverse effects reported in clinical trials; dose-related tolerability limitations.	Frequently combined with silymarin, monacolins, policosanol, curcumin, chromium, hesperidin.	[44,45,46,47]
Banaba (*Lagerstroemia speciosa*; corosolic acid)	Doses range from 10 mg purified corosolic acid to 300 mg extract; non-standardized phytocomplexes.	Limited long-term safety data; unclear contribution of corosolic acid vs. ellagitannins.	Frequently tested in multi-ingredient combinations.	[48,49,50]
*Gymnema sylvestre* (gymnemic acids)	Very heterogeneous dosing (250 mg extract to 6 g leaf powder); different extract types (GS4, OSA).	Limited modern safety profiling; long-duration stimulation of β-cells not extensively evaluated.	Variability in extract preparation and standardization.	[51,52]
*Ilex paraguariensis* (yerba mate)	Preparation-dependent dosing (tea vs. extract vs. capsule); active dose not clearly defined.	Caffeine content may limit use in some populations; safety is dependent on preparation.	Beverage-based interventions reduce standardization; combination tablets include mulberry and chromium.	[53,54,55]
*Ascophyllum nodosum/Fucus vesiculosus*	Doses are 500 mg–2 g; often used in phytocomplex with chromium.	Iodine content may influence thyroid function.	Frequently combined with chromium picolinate.	[56,57,58]

**Table 3:** Micronutrients and bioactive compounds with evidence on glucose metabolism.

Micronutrient/Bioactive Compound	Main Mechanisms	Clinical Evidence	Documented Metabolic Outcomes
Vitamin D	VDR activation; modulation of insulin secretion; reduction in inflammation	Meta-analyses of RCT studies in subjects with prediabetes	↓ risk of progression to diabetes ~10%; ↑ regression to normoglycemia ~24% [59,60,61,62,63,64,65,66,67]
Chromium	Maintenance of glucose homeostasis	Meta-analysis of RCT studies	↓ fasting plasma glucose; no effect on HbA1c [68]
Total antioxidant capacity	Antioxidant activity; modulation of postprandial response	Dose–response meta-analysis (n = 170,919)	↓ risk of prediabetes and diabetes [69]
Total flavonoids	↑ nitric oxide; improved endothelial function	Prospective meta-analyses (>460,000 subjects)	↓ all-cause and cardiovascular mortality; ↓ T2DM risk; insulin sensitivity [70,71,72]

**Table 4:** Plant-Derived Phytocomplexes and Their Effects on Glucose Metabolism: Molecular Targets and Mechanisms.

Phytocomplex	Main Bioactive Compounds	Molecular Targets	Mechanisms of Action
***Mangifera indica*** **(mango)**	Mangiferin, xanthones	AMPKα, SIRT1, PGC-1α	↑ insulin sensitivity; ↑ mitochondrial function; “metformin-like” effect [84,85,86,87,88,89,90]
***Momordica charantia*** **(bitter melon)**	Saponins, momordicin	AMPK, GLUT4, TAS2R, α-glucosidase	↓ carbohydrate absorption; ↑ peripheral glucose uptake; ↑ GLP-1 and satiety [91,92,93]
***Malus domestica*** **(apple)**	Procyanidins, flavonols, phloridzin	SGLTs, inflammatory cytokines, endothelium	↓ postprandial glycemic response; ↓ metabolic inflammation; ↑ endothelial function [94,95,96,97]
**Combination**	Standardized phytocomplexes	AMPK–SIRT1–GLUT4–GLP-1	Synergistic effects on glucose digestion, absorption, and utilization [83]

## Data Availability

No new data were created or analyzed in this study. Data sharing is not applicable to this article.

## Abbreviations

The following abbreviations are used in this manuscript:

T2DM	Type 2 Diabetes Mellitus
MAFLD	Metabolic Dysfunction–Associated Fatty Liver Disease
ADA	American Diabetes Association
IFG	Impaired Fasting Glucose
IGT	Impaired Glucose Tolerance
WHO	World Health Organization
ISTAT	Italian Institute Of Statistics
BES	Equitable And Sustainable Well-Being
GBD	Global Burden Of Disease
IR	Insulin Resistance
SGA	Small For Gestational Age
IUGR	Intrauterine Growth Restriction
PCOS	Polycystic Ovary Syndrome
OGTT	Oral Glucose Tolerance Test
VDRs	Vitamin D Receptors
RCT	Randomized Controlled Trial
TAC	Total Antioxidant Capacity
EFSA	European Food Safety Authority
HOMA-IR	Homeostatic Model Assessment for Insulin Resistance
SGLTs	Sodium–Glucose Transporters
TyG index	Triglyceride–Glucose Index
GP	General Practitioner
